# Development of the Interdisciplinary and Interprofessional Course Concept “Advanced Critical Illness Life Support”

**DOI:** 10.3389/fmed.2022.939187

**Published:** 2022-07-14

**Authors:** Mark Michael, Henning Biermann, Ingmar Gröning, Martin Pin, Philipp Kümpers, Bernhard Kumle, Michael Bernhard

**Affiliations:** ^1^Emergency Department, University Hospital, Heinrich-Heine-University, Düsseldorf, Germany; ^2^Department for Acute and Emergency Medicine, University Hospital RWTH Aachen, Aachen, Germany; ^3^Emergency Department, Krankenhaus Maria-Hilf, Krefeld, Germany; ^4^Interdisciplinary Emergency Department, Florence-Nightingale-Krankenhaus, Düsseldorf, Germany; ^5^Department of Medicine D, Division of General Internal and Emergency Medicine, Nephrology, Hypertension and Rheumatology, University Hospital Münster, Münster, Germany; ^6^Emergency Department, Schwarzwald-Baar Klinikum, Villingen-Schwenningen, Germany

**Keywords:** critically ill patients, emergency medicine, resuscitation room, emergency department, course concept

## Abstract

The Advanced Critical Illness Life Support (ACiLS) course was developed on behalf of the German Society for Interdisciplinary Emergency and Acute Medicine (DGINA). The goal of the ACiLS course is to provide a nationally recognized and certified life support course that teaches medical professionals the key principles of initial care of critically ill patients in the emergency department, including the (PR_E-)AUD^2^IT-algorithm. It is designed for interdisciplinary and multi-professional staff in the resuscitation room to optimize patient safety and outcome. ACiLS includes a new blended learning concept with a theoretical part as comprehensive e-learning and a two-day practical part with strong focus on team training in scenarios and workshops. The course format was conceived to balance best teaching practices within the limited instructional time and resources available. This article describes the development of the ACiLS course and provides an overview of its future implementation.

## Background and Rationale for the Educational Activity Innovation

Critically ill patients are stabilized by emergency medical services (EMS) and subsequently admitted to emergency department (ED) (1, 2). Early recognition of the underlying medical problem and appropriate treatment after arrival at the ED is a prerequisite in order to improve the outcome. Like the Advanced Trauma Life Support (ATLS^®^) and the European Trauma Course (ETC^®^) for severely injured patients, critically ill patients with non-traumatic conditions are often transferred directly to the resuscitation room as a dedicated treatment area in a German ED (1).

The non-trauma critically ill patients treated in the ED's resuscitation room have a wide variety of underlying diseases (1). In contrast to trauma patients (3, 4), information on the epidemiology, early in-hospital care, and diagnostic procedures for non-trauma critical ill patients in the ED is sparse (1, 5). There is evidence that critically non-trauma patients are more often treated than trauma patients in the resuscitation room [relation 4:1 (6)], and these patients have an approximately 3-fold higher mortality than trauma patients who receive care in the trauma bay (1, 5).

Some studies have recently presented data on the initial care of non-trauma critically ill patients (1, 5). The wide spectrum of underlying diseases is distributed according to ABCDE-problems as follows: 3, 29, 35, 32, and 1%, respectively (1, 5). The average duration of treatment in the resuscitation room was 33 ± 23 min and a relevant number of patients required intensive care treatment in the ED due to delayed transfer to an intensive care unit (ICU). Various critical interventions take place in the resuscitation room. Over 80% of these patients were admitted to the ICU. The 30-day mortality rate was 34–36% (1, 5).

According to the results of a recent survey among medical directors of German EDs (7), 84% stated that a specialized training program for non-trauma critically ill patients for physicians and nurses working in German EDs is urgently needed. Already established course concepts like ATLS^®^ and ETC^®^ are specialized for trauma patients, others like Advanced Life Support (ALS), Advanced Cardiac Life Support (ACLS) or Advanced Medical Life Support (AMLS^®^) do not focus on resuscitation room care.

The course concept “Advanced Critical Illness Life Support” (ACiLS) is developed on the initiative of the German Society of Interdisciplinary Emergency and Acute Medicine (DGINA) to structure the care of these patients and to implement a uniform course concept in addition to the existing life support courses in Germany.

## Pedagogical Framework and Principles of the ACiLS Course Concept

### Aims and Objectives

The aim of the ACiLS course is to teach healthcare professionals the key principles of acute care for critically ill patients in an interdisciplinary and multi-professional team approach that reflects the current way critically ill patients are treated in the resuscitation room in German EDs.

The content should cover the immediate differential diagnosis and therapy of the most important and most frequent tracer diagnoses in critically ill non-trauma patients as well as theoretical, practical and communicative skills.

The (PR_E-)AUD^2^IT algorithm (8), a standardized and easy-to-learn concept for the treatment of critically ill non-trauma patients, should be used as a backbone and key element of the course concept.

### Targeted Professionals

The ACiLS course is primarily aimed at physicians and nurses from all specialties who are involved in the management of critically ill non-trauma patients in the ED's resuscitation room.

### Concept and Development

At the beginning of 2020, the project group' ACiLS course concept' was formed within the working group “resuscitation room” of the DGINA. The project group consisted of a total of 40 physicians and nurses with high expertise in clinical emergency medicine and especially resuscitation room care. All aspects of the developed course concept were approved iteratively in numerous consensus conferences by the entire project group. A core group coordinated the global course development, several subgroups for scenarios, workshops and the e-learning part were founded and brought together to periodical online meetings. Despite the current corona pandemic, all participants met under appropriate safety precautions in October 2021 for a rehearsal of the practical part of the course. Table 1 descripted the course development according to Kern's 6-step approach (9, 10).

**Table 1:** “Advanced Critical illness Life Support” course curriculum development using Kern's 6-step framework.

**Kern's 6-step framework**	**“Advanced critical illness life support” course**
**1. Problem identification and general needs assessment**	•critical ill patients with a high mortality risk are treated on a regular basis in ED •training concepts for the treatment of non-traumatic critically ill patients in ED are generally lacking.
**2. Targeted needs assessment**	•no formal course concepts for non-traumatic critically ill patients have been published for staff of Emergency Departments.
**3. Goals and objectives**	•interprofessional ED staff will be able to cover the immediate differential diagnosis and therapy of the most important and most frequent tracer diagnoses in critically ill non-trauma patients •interprofessional ED staff will learn theoretical, practical and communicative skills. •interprofessional ED staff will manage critically ill patients according to the (PR_E-)AUD^2^IT algorithm.
**4. Educational strategies**	•precourse 12 1-h-modules provide the theoretical background, scientific foundations and practical knowledge of management in critically ill patients (diagnosis, clinical reasoning, basic algorithm, principles of crew resource management) using texts, interactive learning material and videos. •facultative lessons lead to deeper understanding and considering the various experience of the target group. •practical part of the course lasts two full days and includes eleven brief lectures, one faculty presented demonstration of the (PR_E-)AUD^2^IT-algorithm, 16 hands-on scenarios with relevant life-threatening emergencies in a ED's resuscitation room setting, and 6 workshops on skills, communication and procedures.
**5. Implementation**	•target learners are physicians and nurses from all specialties who are involved in the management of critically ill non-trauma patients in the ED's resuscitation room. •at least 4 weeks preceeding the face-to-face course, participants receive the online e-learning access •2 day face-to-face course. •at the end of the second day, candidates undergo a written final examination.
**6. Evaluating the effectiveness of the curriculum**	•pre-course e-learning is completed by a multiple-choice test after finishing all parts (passing limit: 70% of correct answers). •permanent assessment of the candidates during the course. •written final examination at the end of the course.

*ED, Emergency Department*.

### Course Components

The ACiLS course is an interprofessional blended learning format that provides a standardized management concept in a team approach. Nurses and emergency physicians run through an e-learning-part consisting of 12 modules for theoretical content, which is completed by a pre-course exam and is scheduled at least 4 weeks before attending the 2-day face-to-face course. As a result of this split-up, the in-person part of the ACiLS course has a very high proportion of practical experience, yet does not neglect the theoretic foundation of emergency medicine.

The 2-day practical part includes, among other things, impulse lectures, team training in small groups based on simulated cases/scenarios, and the teaching of modern emergency procedures at appropriate skill stations.

### The (PR_E-)AUD^2^IT-Algorithm

The (PR_E-)AUD^2^IT-algorithm (8) is the central didactic element of the course system and offers a mnemonic for a structured diagnostic and treatment (Table 2 and Supplementary Figure S1). As is well known from trauma that a structured algorithm for care in the resuscitation room improves the outcome (3). Therefore, the (PR_E-)AUD^2^IT algorithm was developed, incorporating proven components such as the “ABCDE” - approach, primary and secondary survey, fixed reassessments, and others. The implementation of the ACiLS course system and the (PR_E-)AUD^2^IT algorithm will be evaluated in the future, especially in terms of survival of non-traumatic critically ill patients, to verify the benefit of structured resuscitation room management for non-traumatic critically ill patients. Even if it is not yet finally planned, a scientific approach in the outcome comparison may be a before-and-after-study in a given time interval after introduction of the (PR_E-)AUD^2^IT algorithm in monocentric or multicenter investigations in non-traumatic critically ill patients. The concrete implementation of the scientific investigation is currently still part of a continuous development in this field.

**Table 2:** The (PR_E-)AUD^2^IT-algorithm as backbone of the resuscitation room management of critically ill patients.

**Item**	
**P**	Preparation		Information of team memberships about the announced emergency. Preparation of the resuscitation room, check of equipment, personal protective equipment.
**R**	Ressources		Prior information of computed tomography, blood bank, endoscopy, completing the resuscitation team if need be.
**_**	**Team time out I**		During the time out, tasks and responsibilities are clarified, initiate a friendly and respectful welcome of the EMS team, “5-second-round” (“First impression”), structured hand-over.
**E**	Early resuscitation (primary survey)		Early in-hospital resuscitation according to < c>ABCDE and treating life-threatening conditions in order to resuscitate the vital functions. Also known as “primary survey”.
**-**	**Team time out II**		Concludes the early resuscitation phase. Establishing a common understanding of the patient status. Planning the next steps (AUD^2^) which are also known as “secondary survey”.
**A**	Acute medical history	secondary survey	Identification of the leading complaint, evaluation using the SAMPLER scheme [symptoms, allergies, medication, past medical history, last meal, (triggering) event, risk factors] and symptom assessment using the OPQRST scheme (onset, palliation/provocation, quality, radiation, severity and time).
**U**	Urgent examination		Priority-oriented examination according to the leading complaint to rule-out the need for immediate intervention: 12 lead-electrocardiogram by chest pain, focused assessment using ultrasound, re-assessment of < c>ABCDE (critical bleeding, airway, breathing, circulation, disability, exposure).
**D** ^2^	Differential diagnoses		Using leading symptom oriented diagnostic card for identification and evaluation of differential diagnoses in order to reduce missed diagnoses/injuries.
	Diagnostic procedures		Using apparative diagnostics (e.g., laboratory investigation, blood gas analysis, 12 lead-electrocardiogram, extended ultrasound, chest x-ray, computed tomography) for rule-in and rule-out the differential diagnoses.
**I**	Interpretation (**Team time out III**)		Combination of acute history, urgent examination and diagnostics leads to working diagnosis. Planning ahead the next steps in resuscitating or diagnosing.
**T**	To do		Using the guidelines for specific identified diseases, planning further in-hospital course, admission at ICU or normal ward, hand-over, debriefing.

## Pedagogical Format

### Part I: E-Learning for Preparation

After registration, the participant receives access data for the asynchronous e-learning. At least 4 weeks preceeding the face-to-face course, the participants have to work on 12 modules. The 1-h-modules provide the theoretical background, scientific foundations and practical knowledge of management in critically ill patients (diagnosis, clinical reasoning, basic algorithm, principles of crew resource management) using texts, interactive learning material and videos. They come along with facultative lessons that lead to deeper understanding and considering the various experience of the target group. All lessons consider current guideline recommendations and refer to corresponding links.

In addition, participants are introduced to leading symptom-oriented diagnostic cards specially developed for the ACiLS course. A hard copy of the card set is intended to support decision-making in the simulated cases (Supplementary Figure S2).

The e-learning is completed by a multiple-choice test after finishing all parts (passing limit: 70% of correct answers). Successful participation of the e-learning is mandatory for the in-person part of the course, but the participants are allowed to take unlimited attempts. The content of the e-learning chapters is given in more detail in Table 3 and Supplementary Figure S3.

**Table 3:** E-learning part of the “Advanced Critical illness Life Support” course.

**Chapter**	**Issue**	**Content (in addition to learning points, recognition, and differential diagnosis)**	**Duration (min)**
1	**Introduction**	Learning points, aim of the course, trauma and non-trauma resuscitation room management, current studies and treatment concepts, end-of-life-decision.	60
2	**(PR_E-)AUD** ^**2**^ **IT**	(PR_E-)AUD^2^IT-algorithm, preparation, resources, hand-over, early resuscitation, time-time-out, urgent examination (primary survey), differential diagnoses (using symptom-oriented diagnostic cards), interpretation, and todo.	60
3	**Crew Resource Management**	Communication, error management, human factors, 5-s round, “10-for-10,” structured (de)briefing, team work, team approach, leadership, and membership.	60
4	**Airway Management**	Mask ventilation, laryngeal tube, orotracheal intubation, cricothyrotomy, airway management algorithm, concepts of emergency anesthesia for critically ill patients.	60
5	**Dyspnea**	Interpretation of blood gas analysis, invasive and non-invasive ventilation, POCUS of the lungs, needle decompression, and chest tube.	60
6	**Shock**	Hemodynamic monitoring, catecholamines and fluid resuscitation, ACiLS RUSH-protocol.	60
7	**Chest pain**	ECG basics, Cardioversion / defibrillation, focused echocardiography, differential diagnoses of chest pain.	60
8	**Sepsis**	Early identification of sepsis, definition of sepsis and septic shock, 1-h-bundle, search for sepsis source, and antibiotic use.	60
9	**Abdominal pain**	Emergencies presenting with acute abdominal pain and peritonitis, imaging strategies including POCUS, pain control, interdisciplinary management.	60
10	**Unconsciousness / D-problems**	Neurological vs. non-neurological causes, neurologic exam in unconscious patient, differential diagnoses and treatment, and considerations for protective intubation.	60
11	**Intoxications**	Toxidromes, reasonable drug screening, antidote treatment, monitoring, temperature management.	60
12	**Endocrine and metabolic emergencies**	Clinical presentation, pathophysiology, lab analysis, differential diagnoses, and recompensation / substitution.	60

*ECG, electrocardiogramm; RUSH, Rapid Ultrasound in Shock and Hypotension; POCUS, Point of care ultrasound*.

### Face-To-Face Course: 2 Days of ACiLS

The practical part of the course lasts two full days and includes eleven brief lectures, one faculty presented demonstration of the (PR_E-)AUD^2^IT-algorithm, 16 hands-on scenarios with relevant life-threatening emergencies in a ED's resuscitation room setting, and 6 workshops on skills, communication and procedures (Table 4). Cardiopulmonary resuscitation is not part of the course, since this topic is in detail addressed in other courses like the ALS course.

**Table 4:** Pedagogical elements of the “Advanced Critical illness Life Support” course.

11 lectures	10 min Total time: 110 min
1 faculty demonstration	30 min
16 Scenario trainings ∙ Non-trauma scenarios ∙ Debriefing	30 min (first session 15' add-on) Total time: 495 min
6 workshops ∙ Non-invasive ventilation ∙ Patient handover ∙ Structured debriefing ∙ Pericardocentesis ∙ Electrical cardioversion/ 14pttranscutaneous pacing ∙ Ultrasound	4 x 25 min, 2 x 45 min Total time: 190 min
Final assessment ∙ Multiple Choice-test	45 min

The course is designed for 24 candidates (in four groups), and a minimum of eight instructors. The lectures with 110 min (12% of instruction time) represent only a small part of the in-person part, and repeat and emphasize the central points of the e-learning part. The scenario trainings and workshops cumulate to 625 min (66% of instruction time) and are the core educational elements of the ACiLS course (Table 5).

**Table 5:** Course schedule “Advanced Critical illness Life Support” course.

**Day I**	**Day II**
08:00–08:20 Introduction 08:20–08:55 Lectures Basic principles/(PR_E–)AUD^2^IT/Leading symptoms 08:55–09:25 Faculty demonstration 09:25–09:30 Introduction to material 09:50–10:00 Lecture “Dyspnea” 10:00–11:15 Scenario training “Dyspnea” 11:15–11:40 Workshop “Ventilation” 11:40–11:50 Lecture “Shock” 11:50–12:00 Lecture “Sepsis” 13:00–14:00 Scenario training “Shock” 14:00–14:25 Workshop “Patient Takeover” 14:25–14:50 Workshop “Ultrasound 14:50–15:50 Scenario training “Sepsis” 16:10–16:20 Lecture “Acute abdomen” 16:20–17:20 Scenario training “Acute abdomen” 17:20–17:45 Workshop “Debriefing” 17:45–18:00 Course closure day I	08:00–08:10 “Meet your mentor” 08:10–08:20 Lecture “Chest pain” 08:20–08:30 Lecture “Reduced vigilance” 08:30–09:30 Scenario training “Chest Pain” 09:30–10:15 Workshop “Pericariocentesis” 10:35–11:35 Scenario training “Reduced vigilance” 11:35–12:20 Workshop “Arrhythmia” 12:20–12:30 Lecture “Endocrinology” 13:30–14:30 Scenario training “Endocrinology” 14:30–15:30 Scenario training “Miscellaneous” 15:30–15:40 Lecture “Golden principles” 15:50–16:35 Final assessment 17:00–17:15 Course closure day II

Each scenario training and workshop involves an instructor-participant ratio of 1:3. Of the six participants, one takes the role of a team leader, and the remainder assume the other three basic team member positions (two nurses, one physician). Two remaining participants act as observers. Roles are rotated in each scenario so that each candidate has the opportunity to take each position within the team.

Each scenario and workshop includes a team briefing, a non-trauma case with a life-threatening emergency, including application of the (PR_E-)AUD^2^IT-algorithm, and a subsequent feedback as a learning conversation. The work off of the (PR_E-)AUD^2^IT-algorithm addresses management, communication, and leadership issues common to resuscitation of critically ill non-trauma patients. Practice is given in prioritizing resuscitation efforts, delegating tasks, recognizing potential warning signs (red flags), and communicating with other relevant disciplines.

Each scenario is concluded with feedback from the instructors, where learning points and team performance are reviewed. This modular, scenario and workshop-based training represents a new and innovative development in medical education, providing instructions in the core principles of resuscitation and the associated management of critically ill non-trauma patients.

After each simulated scenario, the trainers facilitate a detailed debriefing in which the key learning points are discussed using the diagnosis cards. The debriefings also analyze the communication and interaction between the team members based on the principles of crew resource management.

As demonstrated in the OBSERvE studies (1, 5), patient's handover, initial stabilization, and emergency skills (e.g., airway management, ventilation, ultrasound) are commonly encountered in resuscitation room management. The workshops were designed based on these commonly requested emergency skills to prepare participants and provide a basic knowledge for all professionals. Noninvasive ventilation, patient's handover, ultrasound (modified RUSH protocol), electrical cardioversion and transcutaneous pacemaker therapy, and structured debriefing are topics covered in the workshops of the ACiLS course, which are a sampling of commonly used skills in the resuscitation room. Resuscitation training is explicitly not included, as other course concepts such as ALS or ACLS focus on cardiopulmonary resuscitation and periarrest situations. The ACiLS course can be seen as a complementary qualification to ALS or ACLS, with the two courses having different emphases.

### Assessment of Candidates

The instructors continuously assess the participant‘s progress throughout the whole course within the scenario trainings and the workshops to provide personalized feedback. This continuous assessment allows instructors to identify the need for additional support and individual needs, and supports the participants to learn at their own pace. Personal mentoring is maintained throughout the course and supplemented by short meetings within the groups (mentoring groups). At the end of the second day, the candidates undergo a written final examination.

### Material

All scenarios and workshops use resuscitation manikins (AmbuMan^®^ Advanced, Ambu, Bad Nauheim, Germany) in combination with a simulation software (Skillqube^®^, Skillqube, Rauenberg, Germany) and the usual equipment available as identified in a recent survey (7). All material for the scenarios and workshops is standardized and presented using tablets (iPAD, Apple Inc., Germany).

The e-learning software is based on ARTICULATE 360 (Articulate Global, LLC) and organized by Notfall-Campus.de, the education site of DGINA e.V. Germany.

### Language

The e-learning online part and the teaching material to date is written in German. The event language is German.

### Implementation and Future Perspective

In June 2022, the core group set as potential future instructors will run through a test course with three instructors and 10 participants. A subsequent inauguration course with 10 instructors who attended the test course the days before and 18 participants will be the first real-time ACiLS course, held at the Malteser Bildungszentrum Euregio in Aachen, Germany as an established education center to train all forthcoming instructors.

The further perspective would be expanding to other course centers in Germany from 2023, according to a special catalog of requirements. An international ACiLS course is not yet finally planned, but a corresponding implementation will be evaluated after initial start in Germany.

## Discussion

The present overview is the first description of the interdisciplinary and interprofessional ACiLS course concept, which contains some novel and remarkable features. The blended learning with modular content for theoretical background facilitates an individual learning process and a high proportion of practical education with workshop and scenario training in the two-day face-to-face-course. The course concept unites all members of the resuscitation room management, nurses as well as physicians.

The necessity of developing a separate training concept for non-trauma critically ill patients was firstly published in 2014 (11). There is a great need for a structured training concept both against the background of the patient volume, and from the perspective of those nurses and physician working in the ED (7).

Contrary to trauma management courses [e.g., ATLS^®^ (12–14), ETC^®^ (15, 16)], there are no structured care concepts for critically ill non-traumatic patients in the ED resuscitation room. The ACiLS course is not intended to replace established ALS and AMLS^®^ courses, but to complement them. Rather, ALS courses are a basic requirement for participation in the ACiLS course.

On the one hand, there are courses for teaching basic and advanced measures of cardiopulmonary resuscitation, and on the other hand, there are courses for treating trauma patients (Supplementary Table S1). The AMLS course concept comes closest to our ACiLS course system, but its content is adapted for most out-of-hospital Emergency Medical Service (EMS) systems with training of two-person teams, but not for the resuscitation room of an ED specifically in Germany and not nearly as profound in the acute and emergency care of non-traumatic critically ill patients. It is the deficit of a suitable course concept that goes far beyond existing courses for cardiopulmonary resuscitation with a special focus on non-traumatic critically ill patients that has led to the development of the ACiLS course concept. Even established Crew Resource Management (CRM) course concepts deal intensively with only one aspect of the ACiLS course concept.

The (PR_E-)AUD^2^IT-algorithm (8) is used as a backbone of the structured emergency care concept, however, the algorithm has not been scientifically studied and there are no studies demonstrating a survival benefit. Future studies has to investigate whether patients treated using the (PR_E-)AUD^2^IT-algorithm as a structured treatment and diagnostic pathway receive better care than patients treated without the algorithm.

In conclusion, over a 3-year period, an interdisciplinary and multi-professional group of German specialists in the field of emergency and acute care and education developed a modular training system for critically ill patients. The ACiLS course system includes the (PR_E-)AUD^2^IT-algorithm, uses adult learning techniques, and focusses on scenario and workshop-based training for physicians and nurses. The emphasis on hands-on activities allows for a successful and modern training design, and meets the latest requirements in adult education. The ACiLS course is complementary to established lifesaving courses and is one important piece in the implementation of a nationwide, structured and standardized management of non-traumatic critically ill patients.

## Data Availability Statement

The original contributions presented in the study are included in the article/Supplementary Material, further inquiries can be directed to the corresponding author.

## Author Contributions

Conceptualization, software, and writing–original draft: MM, HB, and IG. Conceptualization and writing–review and editing: MP, PK, and BK. Conceptualization, software, writing–original draft, and funding acquisition: MB. All authors contributed to the article and approved the submitted version.

## Funding

The project received financial support from the German Society of Interdisciplinary Emergency and Acute Care.

## Conflict of Interest

The authors declare that the research was conducted in the absence of any commercial or financial relationships that could be construed as a potential conflict of interest.

## Publisher's Note

All claims expressed in this article are solely those of the authors and do not necessarily represent those of their affiliated organizations, or those of the publisher, the editors and the reviewers. Any product that may be evaluated in this article, or claim that may be made by its manufacturer, is not guaranteed or endorsed by the publisher.

## Supplementary Material

Supplementary Figure S1 (PR_E-)AUD^2^IT-algorithm for the emergency care of critically ill non-traumatic patients in the resuscitation room of an emergency department.

Supplementary Figure S2 Leading symptom orientated card: “Dyspnea”.

Supplementary Figure S3 e-learning chapter No. 2 “(PR_E-)AUD^2^IT”.

Supplementary Table S1 Comparison of course systems.
