# Supplementary material for: Development of the Interdisciplinary and Interprofessional Course Concept “Advanced Critical Illness Life Support”
Source: Front Med (Lausanne). 2022 Jul 14;9:939187. doi: 10.3389/fmed.2022.939187 (PMC9331170; doi:10.3389/fmed.2022.939187)

1 Dyspnea

Basics:

ECG, pulse oximetry, blood pressure, 12-lead-ecg, blood parameters (+ NT-proBNP, creatine kinase, troponine), blood gas analysis, blood glucose, temperature

- ST-elevation myocardial infarction
- ventricular tachycardia
- pulmonary artery embolism
- pulmonary edema
- tension pneumothorax
- pericardial tamponade
- anaphylaxis

(3 POCUS, 6 KU (körperliche Untersuchung))

- acute coronary syndrome
- cardiac arrhythmia
- congestive heart failure
- pleural effusion
- pneumonia
- COPD
- bronchial asthma
- smoke inhalation
- metabolic acidosis

(2 EKG, 3 POCUS, 1a arterielle BGA)

- hyperventilation, psychogenic
- respiratory infections
- severe anemia
- acute paraplegic syndrome
- respiratory disorder
- angioedema
- myasthenia
- aspiration
- rib fracture

(1 Labor+ ven BGA, 4 CT-Diagnostik, 6 KU (körperliche Untersuchung), 5 Röntgen Thorax)

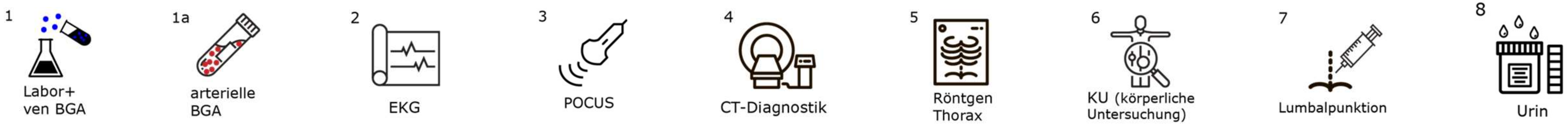

Supplement: Supplementary Figure S2 — Leading symptom orientated card: “Dyspnea”. [file Data_Sheet_2.PDF]
