# Supplementary material for: Development of the Interdisciplinary and Interprofessional Course Concept “Advanced Critical Illness Life Support”
Source: Front Med (Lausanne). 2022 Jul 14;9:939187. doi: 10.3389/fmed.2022.939187 (PMC9331170; doi:10.3389/fmed.2022.939187)
Supplement: Supplementary Figure S3 — e-learning chapter No. 2 “(PR_E-)AUD2IT”. [file Data_Sheet_3.PDF]

Kapitel 02: (PR_E-)AUD²IT (neu)

ACiLS

Lernziele

(PR_E-)AUD²IT – der Basisalgorithmus im nicht-traumatologischen Schockraum

Indikationen

P - Präparation

R - Ressourcen

_ - Team-Time-Out I und Übergabe

E - Erstversorgung (primary survey)

A - Anamnese

U - Untersuchung

D² - Differentialdiagnosen und Diagnostik

I - Interpretation

T - To-Do

Zusammenfassung

Anhang: End-Of-Life-Decision im Schockraum

Literatur

Lernziele

In diesem Kapitel wird Ihnen der Basisalgorithmus des ACiLS-Kurskonzept vorgestellt. Dieser Algorithmus strukturiert die gesamte Schockraumversorgung und nutzt dabei viele bekannte Akronyme und Begriffe, die Ihnen bereits aus der Notfallmedizin vertraut sind. Nach dem Durcharbeiten des Kapitels können Sie:

- …den (PR_E-)AUD^{2}IT Basisalgorithmus des ACiLS-Konzepts beschreiben
- …ein Fallbeispiel unter Zuhilfenahme der in diesem Kapitel beschriebenen Akronyme abarbeiten
- …Bestandteile der Erstversorgung und weiteren Versorgung benennen
- …die Leitsymptomorietierten Differentialdiagnosekarten zur Differentialdiagnostik einsetzen

ACiLS

(PR_E-)AUD²IT-Basisalgorithmus

P – **Präparation:**
- Notfallmedikamente
- Notfallsonographie
- Materialcheck
- Alamierung des Schockraumteams

PSA
- Einmalhandschuhe
- Schutzbrille
- Mundschutz
- ggf. Einmalkittel

R – **Ressourcen:** Information der Transferstellenpartner
- Intensivstation
- Diagnostik (Radiologie)
- Blutprodukte
- Labor

_ – **Team-Time-Out I**
- Vorbereitung abgeschlossen?
- Team vollständig?
- Ersteindruck (5-second round)
- Übergabe

E – **Erstversorgung**

primary survey

A	Airway - freie Atemwege	Monitoring
B	Breathing - Oxygenierung/Ventilation	1 SpO2
C	Circulation - Hämodynamik	2 EKG
		3 NIBD3
D	Disability - Vigilanz	Temp.
		Labor
E	Environment - körperliche Untersuchung und Temperatur	Venöse BGA

- – **Team-Time-Out II**

Beurteile: Zustand stabil vs. kritisch — Formuliere: Leitsymptom/ Prioisierung ABCDE

secondary survey

A	**Anamnese**	SAMPLER, OPQRST
U	**Untersuchung**	Untersuchung und Reassessment
D²	**Differentialdiagnose**	LOD Karte nach Leitsymptomen
	**Diagnostik**	EKG, POCUS, Röntgen, Computertomographie

I – **Interpretation** – **Team-Time-Out III**

Beurteile: Zustand stabil vs. kritisch — Formuliere: Arbeitsdiagnose

T – **To Do**
- Definitive Therapie nach SOP
- regelmäßiges Reassesment

Abb. 1 Basisalgorithmus des Advanced Critical illness Life Support (ACiLS)-Kurskonzeptes

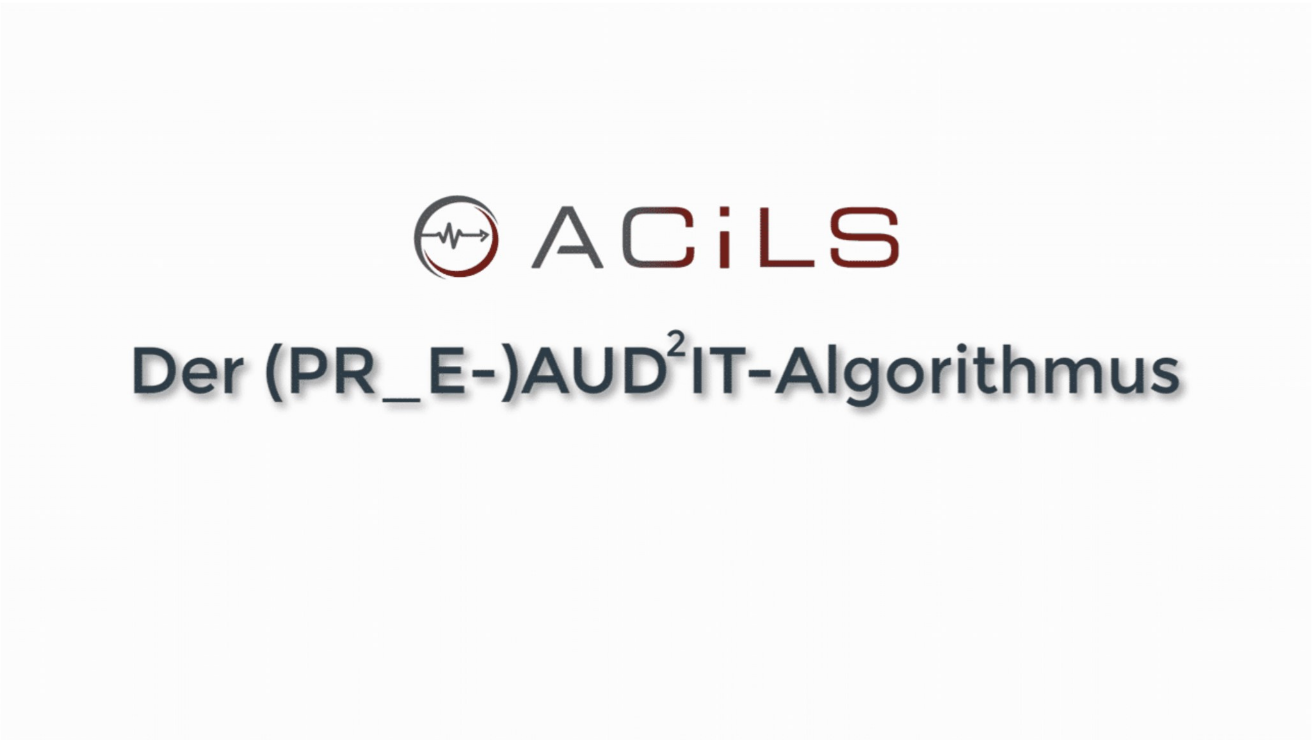

Video 1 Ausschnitt aus dem Lehrfilm *Nicht-traumatologischer Schockraum*: Sequenz (PR_E-)AUD^{2}IT-Algorithmus

Eine Vollversion des Films finden Sie unter: https://www.netzwerk-notaufnahme.de

(PR_E-)AUD²IT – der Basisalgorithmus im nicht-traumatologischen Schockraum

In der prähospitalen und klinischen Notfallmedizin existieren seit Jahren etablierte Untersuchungsschemata in Form von Akronymen, die eine strukturierte Untersuchung und Anamnese beschreiben, so das Vorgehen nach ABCDE, die SAMPLER-Anamnese oder das OPQRST-Schema. In der Trauma-Schockraumversorgung ist das wiederholte Vorgehen nach ABCDE etabliert und in verschiedene Kurskonzepte übernommen worden.

Für die nicht-traumatologische Schockraumversorgung wurde der (PR_E-)AUD^{2}IT-Algorithmus [17] entwickelt, der einen Rahmen für die verschiedene Schritte einer komplexen Schockraumversorgung bietet. Von der Vorbereitung der Schockraumversorgung über die Erstversorgung und Anamnese bis zur Differentialdiagnostik und der Planung weiterer Maßnahmen enthält der Algorithmus alle notwendigen Schritte. Beachtet wird zudem auch die etablierte Struktur sich wiederholender Untersuchungen im Sinne einer Erstversorgung und der weiteren Versorgung, in anderen angelsächsischen Kursformaten als primary- und secondary survey bekannt. Durch die strukturierte Behandlung sollen lebensbedrohliche Zustände rasch erkannt, behandelt und wesentliche Differentialdiagnosen bedacht werden. Strukturierte Versorgungsalgorithmen erhöhen die Patientensicherheit und ermöglichen insbesondere noch wenig erfahrenen Mitarbeitern einen „Leitfaden“ zu nutzen, der in Notfallsituationen die Entscheidungsfindung erleichtert. Aber auch für erfahrene Notfallmediziner bietet der (PR_E-)AUD^{2}IT-Algorithmus einen Leitfaden für eine strukturierte Versorgung und hilft, Fixierungsfehler zu vermeiden. Ziel ist es, dass im Schockraum alle eine "gemeinsame Sprache" sprechen.

In den folgenden Kapiteln sind Sie als Teamleitung im Schockraum aktiv. Dabei steht Ihnen ein engagiertes Team zur Seite, das wir Ihnen jetzt vorstellen möchten.

Ihr Team:

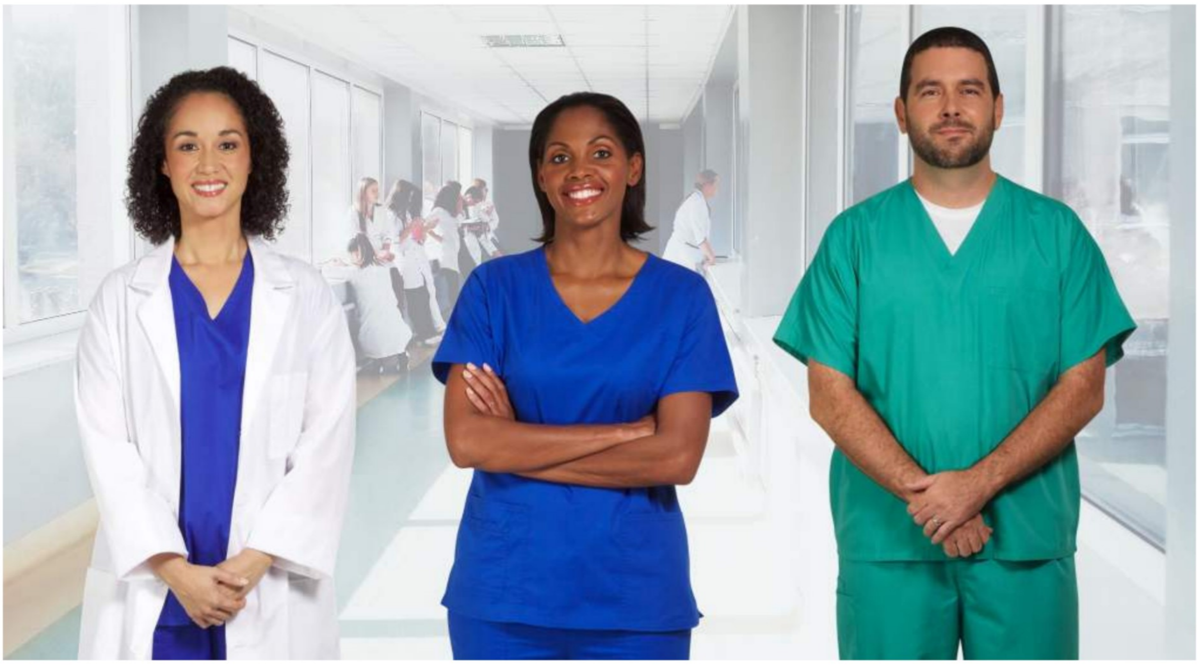

Abb. 2 Ihr Team: Paula, Angela und Michael

Paula, ist Assistenzärztin im 3. Weiterbildungsjahr Innere Medizin. Sie macht gerade ihre zweite Rotation in der Notaufnahme und hat bereits sechs Monate Intensiverfahrung. Paula hat gerade einen ACiLS-Kurs absolviert

Angela, ist seit 20 Jahren Pflegekraft in der Notaufnahme.

Michael, ist Notfallpflegekraft und Rettungsassistent. Er ist seit 8 Jahren hier in der Notaufnahme beschäftigt und ist ALS-Provider und ACiLS-Provider.

Fallbeispiel

Sie sind der Notfallkoordinator der ZNA. Es ist Mittwoch, 11:13 Uhr an einem sonnigen Sommertag, als Ihr Telefon klingelt:

„Hallo, die Notärztin vom 6-NEF-1 hier. Wir sind bei einem 65-jährigen Patienten, der im häuslichen Umfeld reanimationspflichtig wurde. Der Kollaps wurde beobachtet, es bestand keine Latenz, die Tochter hat eine Telefonreanimation durchgeführt. Bei unserem Eintreffen lag ein Kammerflimmern vor, nach etwa 15 Minuten und vierfacher Defibrillation wurde ein ROSC erreicht. Der Patient ist mittels Larynxtubus versorgt und beatmet,

er ist sediert und erhält niedrig dosiert Noradrenalin. Im 12-Kanal-EKG sind keine STEMI-Äquivalente zu erkennen. Ich möchte den Patienten gerne in euren nicht-traumatologischen Schockraum bringen und wäre in 12 Minuten, um 11:25 Uhr bei euch."

Sie fragen die Kollegin noch, ob Beatmungsprobleme vorliegen und wie der Blutdruck ist. Dann bestätigen Sie als diensthabender Notfallkoordinator die Ankündigung des Patienten und lassen die Ankündigung des Patienten und lassen per Funk den „nicht-traumatologischen Schockraum“ alarmieren, denn in Ihrer Notaufnahme versorgen Sie Patieten nach einer Reanimation primär im Schockraum.

Indikationen

Über die Indikation zur Schockraumalarmierung entscheidet der Notfallkoordinator (z.B. der diensthabende ZNA-Oberarzt) nach Voranmeldung durch den Rettungsdienst.

Verschiedene Indikationskataloge sind in den letzten Jahren etabliert und validiert worden (Kapitel 1 – Grundlagen), dies können leitsymptombasierte Indikationen sein („führendes B-Problem"), auf Vitalparameter bezogen sein (SpO_2 <85% unter Sauerstoffgabe, Schocksymptomatik, GCS <10 usw.) oder aber definierte Notfallbilder enthalten (Patient mit respiratorischer Insuffizienz unter nichtinvasiver Beatmungs-Therapie, instabile Bradykardie, Status epilepticus).

In Kliniken unterschiedlicher Versorgungsstufen kann es sinnvoll sein den Alarmierungskatalog an die Bedürfnisse anzupassen. Kleinere Kliniken könnten beispielsweise von einem etwas breiter benannten Indikationskatalog - auch aufgrund des Übungseffekts - profitieren. Eine Orientierungshilfe kann das erwartete *Weißbuch Versorgung kritisch kranker, nicht-traumatologischer Patienten im Schockraum* bieten, das in Kürze veröffentlicht wird. Vor dem Hintergrund einer Über- und Untertriage und der notwendigen Ressourcen ist das Thema Gegenstand intensiver Forschungsbemühungen. Eine abschließende Empfehlung ist auf Basis der aktuell vorliegenden Daten noch nicht möglich.

Folgend ein Besipiel eines Alarmierungsbogens:

Alexianer
ALEXIANER KREFELD GMBH

VA Schockraumalarmierung

1 Ziel

Schaffung einheitlicher Alarmierungskriterien für den **Nicht-Traumaschockraum**

1.2 Abkürzungen

AF – Atemfrequenz
ITN – Intubationsnarkose
GCS – Glasgow Coma Scale
NIV – Nicht-invasive Ventilation
SpO_2 – Sauerstoffsättigung

2 Beschreibung

Zeitpunkt der Meldung: ___:___ Leitsymptom: ____________________
Name: ________________ Geb. Datum: __________ (m / w / d)

Schockraumindikation		
0	☐ Laufende Reanimation<br>☐ Katecholamineinsatz	
A	☐ (Drohende) Atemwegsverlegung<br>☐ Schwere Blutung mit Atemwegsbedrohung	
B	☐ Beatmung (NIV/ ITN) oder High-Flow O_2<br>☐ Initiales SpO_2 unter Raumluft ≤ 85% oder<br>☐ SpO_2 ≤ 89% unter ≥6l O_2<br>☐ AF ≤ 6/min / ≥ 30/min<br>☐ Drohende respiratorische Erschöpfung	
C	☐ Systolischer Blutdruck < 90mmHg trotz Therapie<br>☐ Herzfrequenz ≤ 40/min / ≥ 180/min<br>☐ V.a. Aortendissektion	
D	☐ Akutes neurologisches Defizit<br>☐ Unklare Bewusstlosigkeit<br>☐ Status epilepticus<br>☐ Vigilanzminderung mit GCS ≤ 12<br>☐ V.a. Hirnblutung	
E	☐ Kritischer Gesamtzustand<br>☐ Metabolische Störung trotz Therapie<br>☐ Temperatur ≤ 32°C oder ≥ 40<br>☐ qSOFA ≥ 2 (Kriterien: AF>22, RRsys<100, GCS<15)	

Vitalparameter des Patienten bei Ankündigung:

AF: _____
SO_2: _____
HF: _____
RR _____
GCS: _____
o wach
o ansprechbar
o somnolet
o bewusstlos/ narkotisiert
Temp: _____

Schockraumalarmierung nach Entscheidung Dienstarzt und Patientenzustand

☐ stabil (GELB) **Patienteneintreffen:**

☐ kritisch (ROT) ______________

Abb. 3 Beispiel-Alamierungsplan, Maria-Hilf Krankenhaus Krefeld

UNIKLINIK
RWTHAACHEN
Zentrum für klinische Akut- und Notfallmedizin

Meldebogen – Nicht-traumatologischer Schockraum

Zeitpunkt Meldung: _____:_____ durch: _________________________________ (Tel. siehe umseitig)

Alter: _____ Geschlecht: ☐ w ☐ m

infektiös/Isolation: ☐ ja ☐ nein Grund Iso: ☐ MRE/MRSA ☐ V.a. COVID-19 ☐ andere

Verlegung: ☐ ja ☐ nein aus (KH/Tel.): ___________________________________

Eintreffzeit: _____:_____

A	B	C	D	E
☐ Atemwegsverlegung<br><br>☐ Blutung	☐ Beatmung (NIV/ITN)<br><br>☐ O_2-Bedarf ≥10l/min<br><br>Trotz O_2-Therapie:<br><br>☐ SpO_2 <90%<br><br>☐ AF ≤6/min / ≥30/min<br><br>☐ resp. Erschöpfung	☐ Reanimation/ROSC<br><br>☐ eCPR (Checkliste!)<br><br>☐ Katecholamine<br><br>☐ hämod. relev. HRST<br><br>☐ hämod. Instabilität trotz Therapie<br><br>☐ V.a. Aortendissektion	☐ GCS ≤12<br><br>☐ Status epilepticus<br><br>☐ V.a. ICB/SAB	☐ krit. Gesamtzustand<br><br>☐ metabolische Störung trotz Therapie<br><br>☐ Temp.: ≤32°C / ≥40°C<br><br>☐ Sepsisverdacht
**SAMPLER:**				
**Zusatzinformationen:**				

Alarmierung Schockraumteam (10 min. vor Eintreffzeitpunkt Team zusammenrufen)

Telefonnummern

[illegible]

Abb. 4 Beispiel-Alamierungsplan, Uniklinik RWTH Aachen

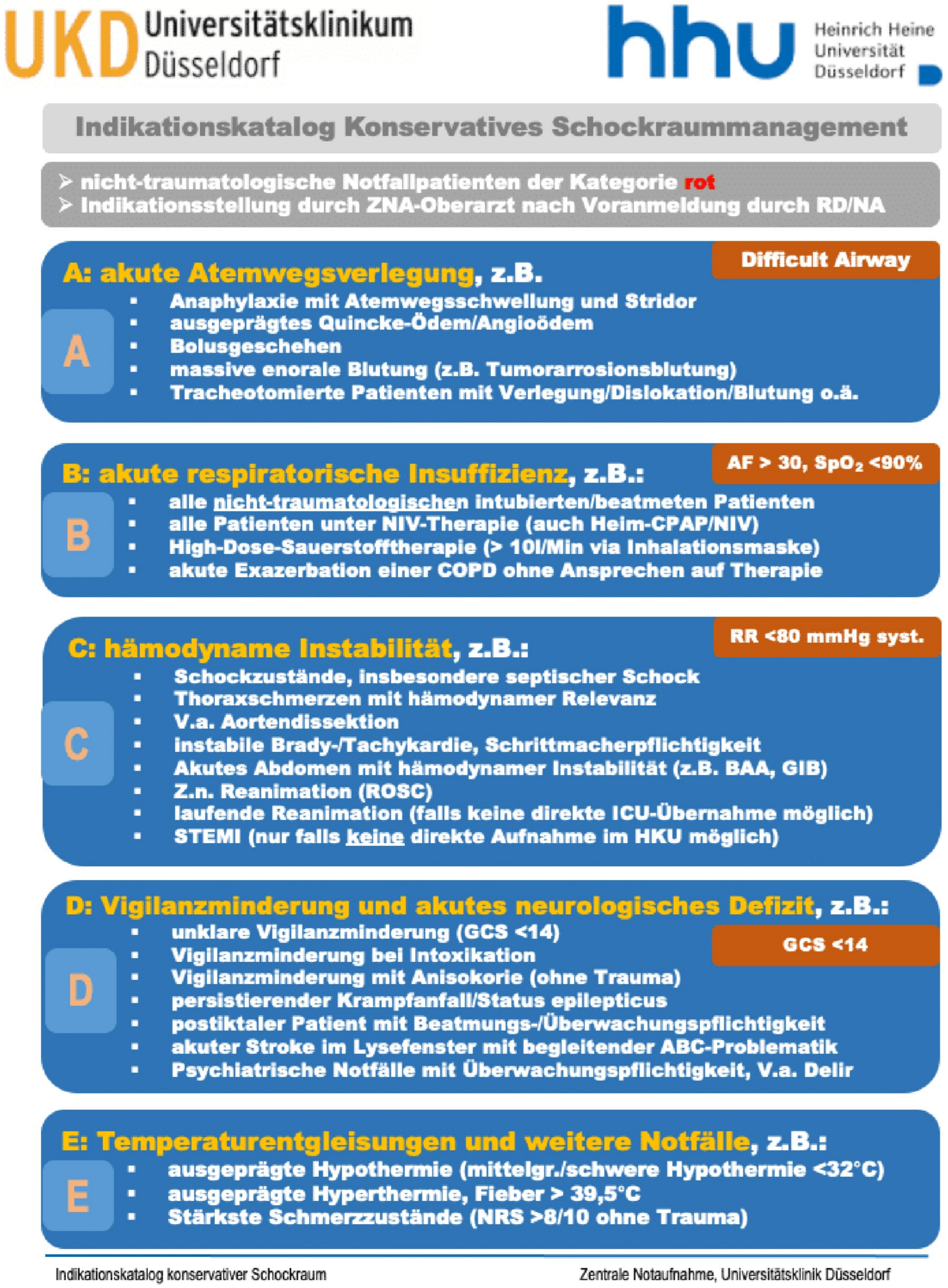

Abb. 5 Beispiel-Alamierungsplan, Universitätsklinikum Düsseldorf

P - Präparation

Zur Vorbereitung des nicht-traumatologischen Schockraums gehört die Alarmierung eines Teams. Die Alarmierung erfolgt in der Regel nach einer **telefonischen Anmeldung durch den Rettungsdienst** oder aufgrund von lokal etablierten Voranmeldesystemen aufgrund der Indikationskriterien. Insbesondere bei kritisch kranken Patienten ist eine (zusätzliche) telefonische Voranmeldung essentiell, um den Zustand des Patienten einschätzen und entsprechende Vorbereitungen treffen zu können. Zudem können so die exakte Eintreffzeit, benötigte Ressourcen und der genaue Übergabepunkt abgestimmt werden. Zur **Dokumentation der Voranmeldung** sollte ein lokal einheitliches Protokoll für den nicht-traumatologischen Schockraum verwendet werden, in dem die relevanten Informationen erfasst werden können.

Die klinikinternen Alarmierungswege variieren je nach Infrastruktur, so kann eine Alarmierung des Schockraumteams über eine Funkschleife oder per Telefon erfolgen, auf jeden Fall sollte ein **standardisierter Alarmierungsweg** definiert werden.

Ein weiterer, **sekundärer Alarmierungsweg** muss auch für Patienten gegeben sein, die entweder im Rahmen der Ersteinschätzung einen kritischen Zustand aufweisen und nicht über den Rettungsdienst zugeführt wurden oder die sich im Laufe der Behandlung in der Notaufnahme klinisch akut verschlechtern. Diese Patienten sollten rasch sekundär in den Schockraum verbracht werden, wo dann die weitere Versorgung stattfindet („**Ad-hoc-Schockraumversorgung**“). Die Alarmierung muss daher abgekürzt auch für diese Fälle etabliert sein.

Nach Alarmierung des **Basisteams** werden im Schritt der **Präparation** die internen Arbeitsmittel der Notfallambulanz und des Schockraums geprüft und vorbereitet.

Neben dem Basisteam (üblicherweise ein Fach-/Oberarzt, ein Assistenzarzt und zwei Pflegekräfte der Zentralen Notaufnahme) können weitere Fachdisziplinen angefordert werden, um ein **erweitertes**

Schockraumteam zu bilden. Abhängig von den verfügbaren Ressourcen, lokalen Strukturen und der angemeldeten, erwarteten Notfallsituation kann dies sein:

- Allgemein-/Viszeralchirurgie
- Anästhesie
- ECMO-Team
- Gefäßchirurgie
- Herz-/Thoraxchirurgie
- HNO
- Kardiologie
- Neurochirurgie
- Neurologie
- und weitere

An dieser Stelle entscheiden Sie sich, das Schockraumteam um einen Kollegen der Kardiologie zu erweitern, da es sich um einen potentiell akut interventionspflichtigen Patienten handelt. Eine telefonische Alarmierung von Stefan, Ihrem für die Zentrale Notaufnahme zuständigen kardiologischen Oberarzt erfolgt durch Sie. Er ist aktuell in einer Untersuchung gebunden, kommt aber so schnell wie möglich in den Schockraum.

Im Rahmen zertifizierter **Cardiac Arrest Center** bilden die Notfallaufnahmen die erste Anlaufstelle in der Klinik, die den geordneten und standardisierten

Übernahmeprozess einleitet. Auch nach den Qualitätskriterien und strukturellen Voraussetzungen von Cardiac Arrest Zentren muss das übernehmende Schockraumteam ("Cardiac Arrest Receiving Team") aus mindestens zwei Ärzten und zwei Pflegekräften bestehen. Dies ist also deckungsgleich zu den Voraussetzungen im nicht-traumatologischen Schockraum [18].

Fallbeispiel

Sie haben sich in der Frühbesprechung im Team abgestimmt und wissen, dass heute mit Ihnen Paula, Michael und Angela im Schockraum eingeteilt sind. Gerade öffnet sich die Tür und die drei treten ein.

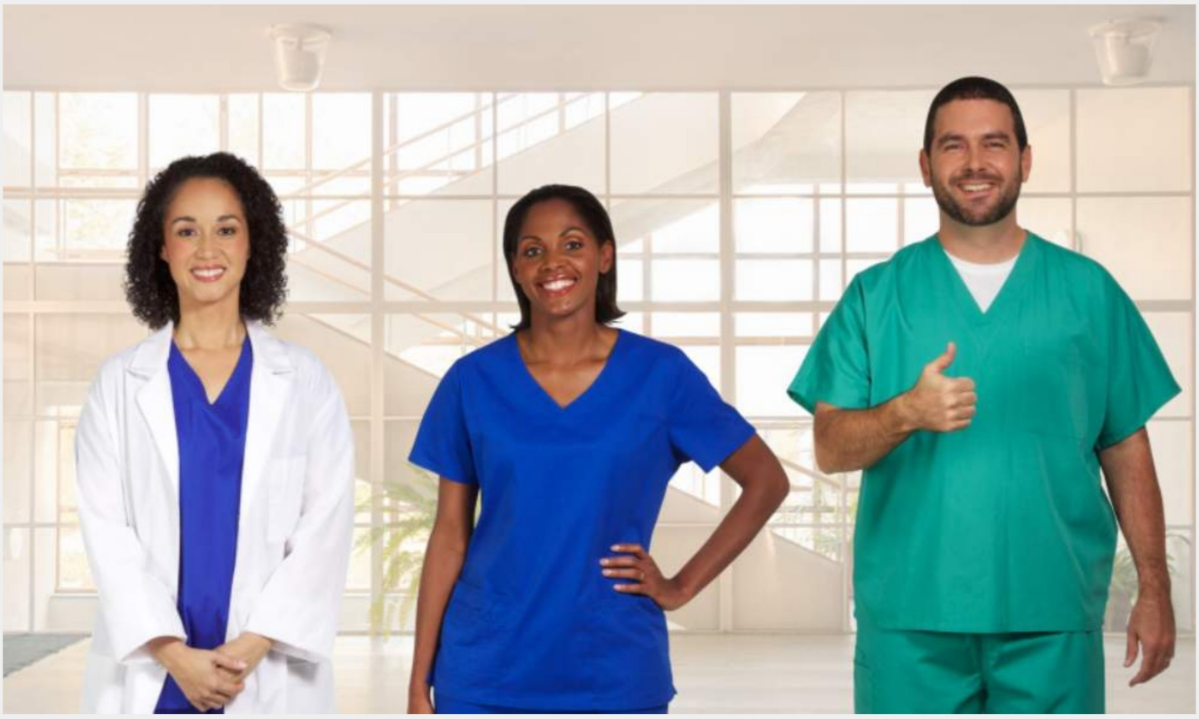

Abb. 6 Ihr Team: Paula, Angela und Michael

Eine gute Vorbereitung ist der Schlüssel, um in der ersten zeitkritischen Phase der Notfallversorgung einen möglichst raschen Überblick gewinnen und den Patienten optimal stabilisieren und behandeln zu können. Der Schockraum sollte dabei stets einsatzbereit gehalten werden. Die Ausstattungsmerkmale wurden bereits in Kapitel 01: Grundlagen behandelt.

Sofort nach Eintreffen der alarmierten Mitarbeiter beginnen Sie mit einem kurzen **Briefing** mit den bekannten Informationen über die erwartete Notfallsituation. Mögliche Besonderheiten werden antizipiert und erwartete Maßnahmen (z.B. Narkoseeinleitung) werden vorbereitet. Es erfolgt eine kurze Vorstellung, damit alle Personen mit Namen angesprochen werden und auch die Qualifikationen im Schockraumteam bekannt sind (folgt in Kapitel 03: CRM). Falls nicht schon vor Eintreffen definiert ist, wer das Team leitet, muss dies als erstes bestimmt werden.

In Ihrer Notfallambulanz ist der Notfallkoordinator als **Teamleitung** gesetzt. Daher teilen Sie im nächsten Schritt das Team ein, um einen erneuten standardisierten **Material- und Gerätecheck** durchzuführen, sowie gemeinsam die **Persönliche Schutzausrüstung (PSA)** anzulegen und den Sitz zu kontrollieren.

Die **Überprüfung und die Vorbereitung des Materials** sollte grundsätzlich in der Phase der **Präparation** erfolgen, um insbesondere nach mehreren Patientenversorgungen die Vollständigkeit von Verbrauchsmaterial und Notfallequipment zu gewährleisten. Auch die Überprüfung von Beatmungsgerät, Defibrillator, Sonographiegerät usw. sollte hier berücksichtigt werden.

In besonderen Fällen ist – je nach Voranmeldung – auch das Zuführen weiterer Geräte für die Diagnostik und Therapie erforderlich, so z.B.:

- Material für den schwierigen Atemweg
- Bronchoskopieturm
- ggf. Material für einen transvenösen Schrittmacher
- Mechanische Reanimationshilfe bei Reanimationssituationen

Weiterhin ist das Anlegen der Persönlichen Schutzausrüstung (PSA, siehe Exkurs im ACiLS E-Learning auf dem Notfallcampus) für das gesamte Team vorgesehen. Hierbei sind Einmalhandschuhe und eine Schutzbrille, Mund-Nasenschutz und je nach Situation FFP2-Maske und Schutzkittel für die Erstversorgung sinnvoll, bis der mögliche Infektionsstatus des Patienten eruiert werden kann. Aufkleber mit Funktionsbeschreibungen erleichtern das Erkennen im Schockraum. Zudem muss insbesondere beim Atemwegsmanagement mit Aerosolbildung gerechnet werden. Dies gilt insbesondere vor dem Hintergrund der COVID-19 Pandemie.

Ihr Team überprüft nun das Beatmungsgerät, bereitet Medikamente für die Sedierung sowie Katecholamine vor. Weiterhin ist das Sonographiegerät bereits vor Ort und es steht eine mechanische Reanimationshilfe bereit, sollte der Patient erneut reanimationspflichtig sein. Während des Material- und Ausrüstungschecks lassen Sie für den Patienten im Krankenhausinformationssystem einen Fall anlegen (z.B. Unbekannt, Non-Traumaschockraum 222/2022). Standardisierte Untersuchungen wie eine Laborentnahme oder antizipierte Untersuchungen wie eine CT-Untersuchung können so bereits vorab angemeldet und zusätzliche administrative Arbeitsbelastung in der Akutversorgung vermieden werden. Sie melden bereits vorab eine Computertomographie („Postreanimationsspirale“) an, da dies in Ihrem Haus zum Standard der Postreanimationsversorgung gehört.

Nach erfolgter Prüfung und Abschluss der Vorbereitung hören Sie die Rückmeldungen von Ihrem Team:

- **Michael**: *„Beatmungsgerät und Defibrillator gecheckt, Intubationstisch komplett.“*
- **Angela**: *„Material ist vollständig, Sufentanil-Perfusor, Propofol-Perfusor, Adrenalin 100 µg/ml und Noradrenalin 10 µg/ml sind aufgezogen.“*
- **Paula**: *„Sono läuft. PSA ist angelegt. Schaust du mal bitte, ob Maske und Brille richtig sitzen?“*
