# Supplementary material for: Development of the Interdisciplinary and Interprofessional Course Concept “Advanced Critical Illness Life Support”
Source: Front Med (Lausanne). 2022 Jul 14;9:939187. doi: 10.3389/fmed.2022.939187 (PMC9331170; doi:10.3389/fmed.2022.939187)
Supplement: Supplementary Table S1 — Comparison of course systems. [file Table_1.DOCX]

| **Supplemental Material Table S1: Comparison of course systems** | | | | | | |
| --- | --- | --- | --- | --- | --- | --- |
|  | **Advanced Critical Illness Life Support** | **Advanced Medical Life Support** | **Advanced Life Support** | **Advanced Cardiac Life Support** | **European Trauma Course** | **Advanced Trauma Life Support** |
| **Abbreviation** | **ACiLS** | **AMLS** | **ALS** | **ACLS** | **ETC** | **ATLS** |
| **Organization** | German Society for Interdisciplinary Emergency and Acute Medicine (DGINA) | National Association of Emergency Medical Technicians  (NAEMT), local medical societies | European Resuscitation Council (ERC) | American Heart Association (AHA) | European Resuscitation Council (ERC) | American College of Surgeons (ACS), local medical societies |
| **Countries** | Germany | United Stated of America, European Union | Europe, Africa, Middle and Far East | United Stated of America, worldwide | European Union | worldwide |
| **Patient collective and setting** | non-traumatic critically ill patients, resuscitation room | emergency patients, out-of-hospital | non-trauma (and trauma) patients suffering from cardiac arrest, out-of-hospital and inhospital, not specific for the resuscitation room | non-trauma (and trauma) patients suffering from cardiac arrest, out-of-hospital and inhospital, not specific for the resuscitation room | trauma patients, resuscitation room | trauma patients, resuscitation room |
| **Duration** | 4 days (2 days  e-learning and 2 days face-to-face-course) | 2 days | 2 days | 2 days | 3 days | 2 days |
| **Target group,** | nurses, physicians | paramedic, nurses, physicians | all healthcare professionals who are expected to provide cardiopulmonary resuscitation in a team | healthcare professionals who either direct or participate in the management of cardiac arrest or other cardiovascular emergencies and for personnel in emergency response | physicians, nurses | mainly physicians (special course concept implemented for nurses 🡪 Advanced Trauma Care fpr Nurses, ATCN) |
| **Structure and educational concept** | theory: pre-course online e-learning and low number of short lectures,  practice by  extended  simulation based training and workshops | theory by course book and lectures, practice by simulation based training | theory by online course,  practice by workshops, skill stations,  lectures, cardiac arrest simulation (CAS) training | theory by video lectures, practice by simulation based training | theory by course book and lectures,  practice by hands-on part, simulation based training and skill stations | theory by course book, lectures, Practice by hands-on part, simulation based training and skill stations |
| **Certification** | yes | yes | yes | yes | yes | yes |
