# Supplementary material for: Development of the Interdisciplinary and Interprofessional Course Concept “Advanced Critical Illness Life Support”
Source: Front Med (Lausanne). 2022 Jul 14;9:939187. doi: 10.3389/fmed.2022.939187 (PMC9331170; doi:10.3389/fmed.2022.939187)
Supplement: Supplementary Figure S1 — (PR_E-)AUD2IT-algorithm for the emergency care of critically ill non-traumatic patients in the resuscitation room of an emergency department. [file Data_Sheet_1.PDF]

ACiLS

(PR_E-)AUD2IT- Basic algorithm

**P**	**Preparation**	**Personal protective equipment**
**R**	**Ressource**	
**_**	**Team-Time-Out I**	First impression (5-second round)
**E**	**Early resuscitation (primary survey)**	

primary survey

- **A** Airway
- **B** Breathing
- **C** Circulation
- **D** Disability
- **E** Environment

**-**	**Team-Time-Out II**<br>Evaluate stable vs. critical	Leading symptom? working diagnosis

secondary survey

**A**	**Acute medical history**	SAMPLER, OPQRST
**U**	**Urgent examination**	Focused assessment
**D²**	**Differential diagnoses**	Identification and evaluation of differential diagnoses
	**Diagnostic procedures**	POCUS, x-ray, computed tomography

**I**	**Interpretation**<br>Evaluate stable vs. critical	**Team-Time-Out III**<br>Leading symptom? working diagnosis
**T**	**To Do**	Further therapy
